# Biological and internal dosimetry for radiation medicine: current status and future perspectives

**DOI:** 10.1093/jrr/rrab119

**Published:** 2021-12-31

**Authors:** Wanwisa Sudprasert, Oleg V Belyakov, Satoshi Tashiro

**Affiliations:** Department of Applied Radiation and Isotopes, Faculty of Science, Kasetsart University, 10900 Bangkok, Thailand; International Atomic Energy Agency, 1400 Vienna, Austria; Hiroshima International Council for Health Care of the Radiation-Exposed, 730-8511 Hiroshima, Japan; Hiroshima University, 734-8553 Hiroshima, Japan

**Keywords:** biological dosimetry, internal dosimetry, dose calculations

## Abstract

The International Atomic Energy Agency (IAEA) and Hiroshima International Council for Health Care of the Radiation-Exposed (HICARE) jointly organized two relevant workshops in Hiroshima, Japan, i.e. a Training Meeting ‘Biodosimetry in the 21^st^ century’ (BIODOSE-21) on 10–14 June 2013 and a Workshop on ‘Biological and internal dosimetry: recent advance and clinical applications’ which took place between 17 and 21 February 2020. The main objective of the first meeting was to develop the ability of biodosimetry laboratories to use mature and novel techniques in biological dosimetry for the estimation of radiation doses received by individuals and populations. This meeting had a special focus on the Asia-Pacific region and was connected with the then on-going IAEA Coordinated Research Project (CRP) E35008 ‘Strengthening of “Biological dosimetry” in IAEA Member States: Improvement of current techniques and intensification of collaboration and networking among the different institutes’ (2012–17). The meeting was attended by 25 participants, which included 11 lecturers. The 14 trainees for this meeting came from India, Indonesia, Japan, Malaysia, Philippines, Republic of Korea, Singapore, Thailand and Vietnam. During the meeting 13 lectures by HICARE and IAEA invited lecturers were delivered besides eight research reports presented by the IAEA CRP E35008 network centers from the Asia-Pacific region. Two laboratory exercises were also undertaken, one each at Hiroshima University and the Radiation Effects Research Foundation (RERF). The second training workshop aimed to discuss with the participants the use of mature and novel techniques in biological and internal dosimetry for the estimation of radiation effects by accidental, environmental and medical exposures. The workshop was attended by 19 participants from Indonesia, Jordan, Oman, Philippines, Singapore, Syrian Arab Republic, Thailand, UAE, USA and Yemen. The main outcome of both meetings was a review of the state-of-the-art of biodosimetry and internal dosimetry and their future perspectives in medical management. This report highlights the learning outcome of two meetings for the benefit of all stake-holders in the field of biological and internal dosimetry.

## INTRODUCTION

Human beings can receive ionizing radiation on a daily basis, either from those occur in nature or man-made radioactive materials. The chance of radiation exposure increases in various cases such as radiation workers, patients that need to be diagnosed or treated with radiation, and those who are in the event of a radiation accident or terrorism, etc. The international agencies involved in the prevention of radiation hazards have issued guidelines for prevention, practices and various measures to reduce the chance of radiation exposure and reduce the potential impact of those cases. The main idea comes from the knowledge of the term ‘absorbed dose.’ (Defined as the mean energy imparted by ionizing radiation to matter in a volume element divided by the mass of matter in the volume element, see Radiation Protection and Safety of Radiation Sources: International Basic Safety Standards (IAEA Safety Standards Series No. GSR Part 3, Vienna, 2014).) In case of patients who need to be diagnosed or treated with radiation, the International Commission on Radiological Protection (ICRP) has issued a manual and requirements for medical radiation to protect both medical workers and patients. However, in case of nuclear and radiation accidents or emergency situations, those involved in the event do not often have personal radiation dosimeters. The first thing that must be done quickly is to distinguish between those who are exposed and are not exposed to radiation in order to provide them with timely medical treatment. Therefore, the amount of radiation the patient receives is very important and necessary for an effective treatment plan.

**Fig. 1.**
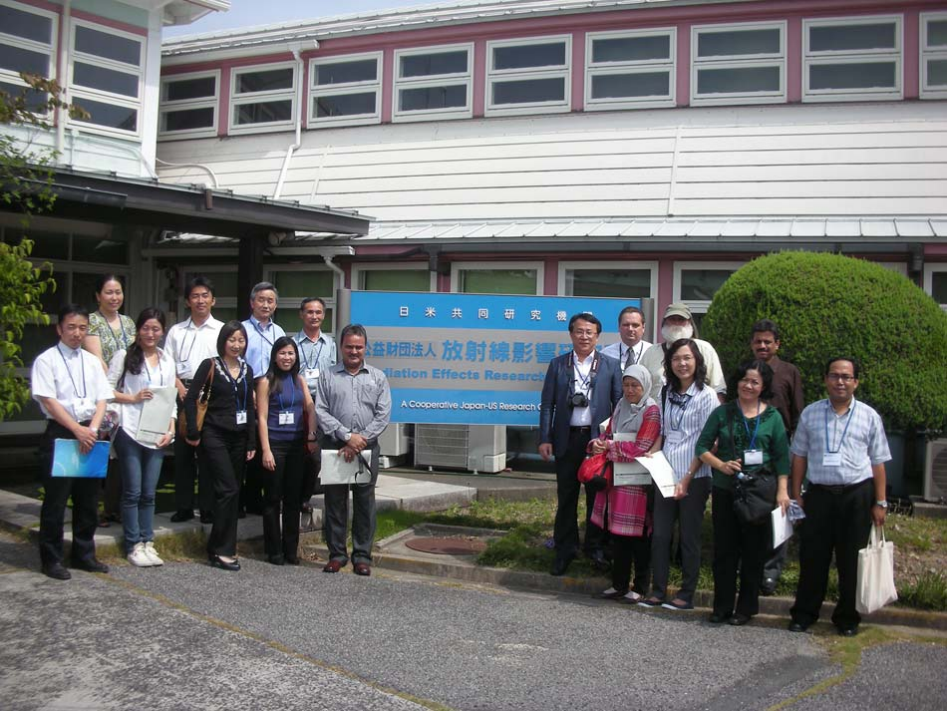
Participants of ‘Biodosimetry in the 21^st^ century’ at RERF (photo: IAEA).

**Fig. 2.**
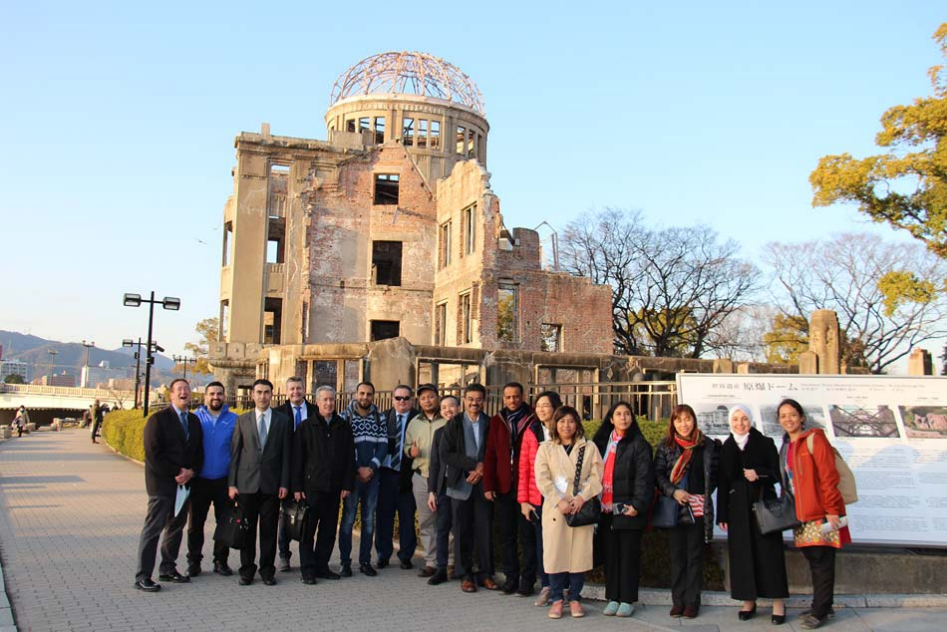
Participants of ‘Biological and internal dosimetry: recent advance and clinical applications’ at The Atomic Bomb Dome (photo: HICARE).

Biological dosimetry has become a valuable tool when person does not have personal radiation measuring devices or when those devices cannot provide important information due to the inter-individual variation in biological response to radiation. In cases of people who work with unsealed radioactive materials, internal exposure occurs when radioactive materials enter the body during routine work or following an incident. The assessment of radiation doses due to internal exposure can be done based on determination of internal contamination, using state-of-the-art models recommended by the ICRP.

In medical uses of radiation, the biological and internal dosimetry becomes important tools to estimate of the amount of radiation that is absorbed by different organs or organ systems. Recently, new biological and internal dosimetry methods with higher sensitivity and throughput, such as gamma-H2AX assays and PNA-FISH analyses, have been established. These new methods could be applied for the appropriate management of medical exposure for diagnosis and treatment, which can lead to individualized patient radiotherapy and/or radiological diagnostic schedules. However, the continuous development of high-throughput biodosimetry techniques are needed not only to deal with large-scale radiological accidents but also to be applied for the effective radiation diagnosis and therapy.

In order to meet these specific objectives, the International Atomic Energy Agency (IAEA) initiated a Coordinated Research Project (CRP) E35008 titled ‘Strengthening of “Biological dosimetry” in IAEA Member States: Improvement of current techniques and intensification of collaboration and networking among the different institutes’ (2012–17). In continuation of this effort, the IAEA organized two relevant workshops in cooperation with the Government of Japan through the Hiroshima International Council for Health Care of the Radiation Exposed (HICARE). The first one titled ‘Biodosimetry in the 21^st^ century (BIODOSE-21)’ was organized in Hiroshima between 10 and 14 June 2013 (see Fig. 1) with the objectives of: (i) review the state-of-the-art of biodosimetry in the IAEA Member States of Asia-Pacific region, and (ii) develop and/or enhance the capabilities of biodosimetry laboratories in the region. The meeting was attended by 25 participants, which included 11 lecturers. The 14 trainees for this meeting came from India, Indonesia, Japan, Malaysia, Philippines, Republic of Korea, Singapore, Thailand and Vietnam. The training meeting provided lectures or presentations followed by adequate opportunity for extensive discussion to foster exchange of ideas and experiences, followed by a laboratory exercise in biodosimetry for all trainees at Hiroshima University and the Radiation Effects Research Foundation (RERF). However, due to the lack of expertise in low- and middle-income countries and to retain expertise in existing individuals, the IAEA launched a CRP E35010 on ‘Applications of Biological Dosimetry Methods in Radiation Oncology, Nuclear Medicine and Diagnostic and Interventional Radiology (MEDBIODOSE)’ in 2017. The second workshop on ‘Biological and Internal Dosimetry: Recent Advance and Clinical Applications’, was organized again in Hiroshima between 17 and 21 February 2020 (see Fig. 2), which was an event under the IAEA technical cooperation project RAS9087, ‘Enhancing Capacity in States Parties in Internal Dosimetry for Occupational Exposure (ARASIA)’. The main objective of the workshop was to discuss with the participants the use of mature and novel techniques in biological and internal dosimetry for the estimation of radiation effects by accidental, environmental and medical exposures. This workshop also addresses a few complementary topics on internal dosimetry including the principles of individual monitoring and internal dosimetry; assessment of internal doses from monitoring measurements; biokinetic and dosimetric models; monitoring and dosimetry for first responders after a major accident; and the application of internal dosimetry to health risk assessment. The workshop was attended by 19 participants from Indonesia, Jordan, Oman, Philippines, Singapore, Syrian Arab Republic, Thailand, UAE, USA and Yemen. Many of the participants were from the same group as those who attended the first workshop.

This report provides a report of two training meeting biological and internal dosimetry arranged by IAEA and HICAREY in 2013 and 2021. *Inter alia*, some relevant recent advances and the future prospects for application of biological and internal dosimetry in management of medical radiation exposure were also discussed.

## THE STATE-OF-THE-ART OF BIODOSIMETRY TECHNOLOGY

Biodosimetry refers to the quantitative estimation of absorbed radiation dose in individuals to help assess the health risks for exposed individuals. Individual or population exposure to radiation might occur due to medical interventions (diagnostic and/or therapeutic), profession (workers in radiation facilities, the nuclear industry, etc.) or large-scale incidents/accidents (e.g. accidents in nuclear industries, nuclear test, fallouts, nuclear terrorism or dirty-bombs). Accurate assessment of the radiation dose in shortest possible time is especially important for successful and effective triage and medical management. One example of such an event in the most recent past is that at TEPCO Fukushima Daiichi Nuclear Power Plant (NPP) in Fukushima Prefecture after the great east Japan earthquake and tsunami on 11th March 2011 [1]. In such large-scale nuclear incidents, biological dosimetry based on cytogenetic assays performed on the peripheral blood lymphocytes (PBL) is normally employed to estimate the absorbed dose in the exposed or suspected-to-be-exposed individuals. Cytogenetic biodosimetric assays are based on a number of biological endpoints essentially related to chromosomal aberrations (CA) in PBL. Such assays assess the radiation absorbed dose based on quantification of radiation induced: (i) dicentric type of chromosomal aberration (DCA), (ii) cytokinesis-block micronuclei (CBMN), (iii) premature chromosome condensation (PCC) fragments/rings, or (iv) fluorescence *in situ* hybridization (FISH) translocation using appropriately developed methods of assays [2]. Three techniques—DCA, CBMN and FISH—have been standardized to date for monitoring and quantifying the resulting CA in PBL in order to estimate radiation absorbed dose. PCC analysis is under active consideration for standardization. Among these, DCA assay in PBL is considered the ‘gold standard’ for biological dosimetry for radiation emergency medicine by IAEA and other international agencies, because of its proven utility in the past large-scale nuclear incidents or accidents such as the Chernobyl accident in 1986 [3, 4], the Goiania accident in 1987 [5], the JCO Tokaimura, Ibaraki Prefecture, Japan, accident in 1999 [6, 7] and the Bulgaria accident in 2011 [8].

However, cytogenetic assays for quantitative estimation of CA suffer from methodological limitations because most of them require a cell culture step, other preparations and scoring under microscopes [2]. Therefore, the time required for performing such biodosimetric measurements becomes longer than desirable. It becomes even more difficult when a very large number of samples need to be processed as is likely in a large-scale nuclear incident. Furthermore, this may also result from exposure to several other genotoxins and stimuli as well. There are also issues of instability, over- or under-estimations of radiation exposure doses and the cost of the assays for different cytogenetic parameters. Despite these limitations, the DCA assay in PBL is currently the most sensitive, reliable and widely used quantitative biomarker of radiation exposure. However, notwithstanding the robust infrastructure and preparedness in Japan, the experience of management of the exposed population of the affected area following Fukushima nuclear disaster in 2011 was an eye-opener [1]. The actual time required to perform biodosimetry on the small section of population in the Fukushima area using DCA assay was far too long to be ideal for triage and medical intervention. Lack of trained personnel and preparedness could further impede the biodosimetric assessment of exposed populations in densely populated regions of the world, including the Asian-Pacific region. In times of such exigencies, assessment of individual dose of exposure in a population should be carried out within the shortest possible time for effective triage and medical intervention. Hence, alternative high-throughput biodosimetry technologies are needed and efforts are required to develop such options to deal especially with nuclear incidents of large magnitude.

The early-response following a nuclear incident includes physical measurement of radioactivity in the exposed individuals and clinical observations [9, 10] including, complete blood counts (CBC) with white blood cell differential count [10–12] and sampling of blood for the CA cytogenetic bioassays for dose assessment [2, 13, 14]. It is important to use an additional bioassay to determine radiation dose using other available dosimetry approaches (e.g. dose assessment by measurement of free radicals in solid matrix materials using electron paramagnetic resonance [EPR] among others) [10, 15, 16]. DCA assay, the gold standard currently, has an internationally standardized protocol for uniformity of dose assessment [2, 17]. When faced with large number of samples for DCA assay, which requires a cell culture time of 48 h and additional time for DCA scoring, 50 metaphases may be scored in the triage-mode and 1000 metaphases or up to 100 dicentric chromosomes in the full-scoring mode. Even then, DCA assay takes on average about 52 h to get a result for medical triaging of a patient. Therefore, in the event of a large-scale radiation incident involving a sizeable population group, application of this gold standard technology of biodosimetry becomes very challenging. In such situations, domestic and international collaborations and technical harmonization are necessary [14]. Development and improvement of automated systems with software-driven dose estimation, including curve-fitting (CABAS and DoseEstimate) and dose calculating modules for whole body, partial body and protracted exposure, has made some difference to the effective application of DCA assay for biodosimetry of large population groups [18, 19]. However, further efforts are still required to develop more rapid dicentric scoring methods. A high resolution, modified DCA assay, could be the peptide nucleic acid-fluorescence *in situ* hybridization (PNA-FISH) based CA scoring [20]. Since PNA probes have higher binding affinities to its complementary DNA, the time required for hybridization is much shorter than the conventional DNA probes. This offers an opportunity to quickly perform biodosimetry using PNA-FISH technology helping timely formulation of the treatment strategies during large-scale radiation emergencies. The dicentric analysis by PNA-FISH is thought to be relatively less expensive making it a possible candidate for the next generation biodosimetry especially for low- and middle-income countries [20, 21].

Another attractive cytogenetic technique of biodosimetry is the PCC assay in which the chromosome formation is artificially induced in the genome of PBL. In the fusion approach, the target interphase cells are fused with Chinese hamster ovary (CHO) cells in its M-stage of the cell cycle after treatment with polyethylene glycol [22]. To apply this biodosimetry technique, the mitotic CHO cells are kept ready in a freezer for eventual use. Alternatively, Gotoh *et al.* [23] performed PCC assay in PBL by chemical method at any stage of the cell cycle after treatment with specific inhibitors of serine/threonine protein phosphatase, such as Okadaic acid and Calyculin A. This chemical method has been applied in biodosimetry to analyse chromosome aberration in form of ring and also dicentric chromosomes, using PNA centromere probes, in human lymphocytes at the G2/M stage. This facilitates quick scoring of the aberrations to estimate radiation dose since the frequencies of ring and dicentric chromosomes are directly proportional to the radiation dose [24]. Cytokinesis-block micronucleus (CB-MN) assay may also be applied in a large nuclear incident. However, it has to be borne in mind that other genotoxic stresses besides radiation also induce DNA fragmentation, which could appear as micronuclei in the cytoplasm of the cell [2, 25–27]. Moreover, the production of micronuclei is gender specific and also shows strong influence of age on the frequency of MN induction in a population [28–30]. Notwithstanding these limitations, the simplicity of scoring and requirement of less technical expertise make CB-MN assay a useful tool of triage biodosimetry in a large-scale nuclear incident.

Whether scoring translocations could be more useful than scoring dicentric chromosomes to estimate total accumulated dose in accidentally or occupationally exposed individuals post-exposure at LDR remains a point of debate. The multiplex fluorescence *in situ* Hybridization (M-FISH) is a technology in which 24-color karyotyping is possible. Though expensive, the assay is sensitive and may be used for dose calculations relatively more accurately. In a study in mouse systems, it has been reported that while the frequencies of translocations and dicentrics increased linearly with dose at a dose rate range of 1 to 20 mGy/day, translocations were three-times more pronounced than dicentrics [31]. The biodosimetry studies performed decades after exposure (also referred to as retrospective biodosimetry) on A-bomb survivors throw some light on this debate. For retrospective biodosimetry, unstable-type aberrations (dicentrics and rings) are not suitable since they progressively disappear with time. Quantitative detection of stable-type aberrations (translocations and inversions) are the only choice in such cases because they persist for decades. Accordingly, DS86-estimated dose has been reported based on studies involving 230 A-bomb survivors using FISH technology for scoring of stable chromosome aberrations [32]. In another approach to retrospective dosimetry, an EPR technique may also be applied for estimation of radiation dose using tooth enamel. Molars donated by A-bomb survivors have been subjected to such estimations of dose yielding similar estimates. The dose estimate derived from the amalgamation of FISH and EPR dosimetry results could be better foundation for the new DSO2 dose estimation. This is perceived to be more accurate than other cytogenetic assays wherein variations might creep in due to several factors including triage measurement protocols—measurement of only 100 (Giemsa) or 500 cells (FISH), measurement error (EPR), observer bias (Giemsa and FISH), medical exposure (both) among others. However, the EPR technique does not allow to assess the (in)homogeneity of the exposure, and the EPR dose is always a local dose to the object (in this case, a tooth), while the cytogenetic dose (even estimated by FISH) is always a dose to lymphocyte population in human body, with the (in)homogeneity detection by default.

To enhance the preparedness to deal with nuclear incidents of large magnitudes, it is equally important to review the past experiences of similar magnitudes, analyse the lacunae prior to and following the incident and fill up the voids, deficiencies and inadequacies. The most recent event of this magnitude was the nuclear disaster at Fukushima Daiichi NPP on 11 March 2011. A natural calamity (a combination of a high magnitude earthquake and tsunami) triggered the incident, which also severely damaged 80% of all hospitals in Iwate, Miyagi and Fukushima areas. Due to this, only a small number of hospitals could provide medical care to the injured populations. Not all of approximately 400 Disaster Medical Assistant Teams (DMATs) (1800 medical personnel) could reach their target sites due to extensive damage to communication infrastructure. The leakage of radioactive gases led to evacuation order from 30-km radius of the Fukushima NPP. Consequently, several primary hospitals in the area became non-functional. The remaining primary hospitals could only partially function due to shortage of medical personnel, severely crippling the radiation-emergency medical system in the critical hours of need. More than 800 patients from different hospitals needed to be evacuated on 14 March 2011 by buses or police vehicles, this took a long time and patients were without medical care during this time. This also resulted in loss of lives of 60 elderly patients due to hypothermia, deterioration of underlying medical problems and dehydration [33, 34]. Hence, the Fukushima experience taught us three important lacunae in management of large-scale radiation incidents: collapse of the local radiation-emergency medical system, loss of life during evacuation and deficiency of information on radiation risks to general population. Experience gained from the biodosimetry effort made by the clean-up workers of Chernobyl following the nuclear accident in 1986 is another event of importance [2–4]. The epidemiological studies with A-bomb survivors in Japan are another pointer to help tide over the lacunae and deficiencies of the past. Achievements and progress of scientists and biodosimetry personnel from eight Asia-Pacific region Institutions collaborating under the IAEA-CRP E35008 (Aims, results and experiences gained during the CRP E 35010 (2012–2017) are described in 19 papers published as a special issue of the open source Genome Integrity Journal 2016–2017 Vol. 7–8, see the editorial at http://www.genome-integrity.org/text.asp?2016/7/1/1/197170.) were discussed. While five Member States—Indonesia, Malaysia, Philippines, Thailand and Vietnam—shared their newly established standard dicentric calibration curves prepared at their new biodosimetry laboratories, three Member States (India [35–37], Republic of Korea and Singapore) straightened their collaboration in terms of sample exchange and sharing new biodosimetric methodologies.

## THE STATE-OF-THE-ART OF INTERNAL DOSIMETRY

Internal dosimetry is the science of internal radiation dose assessment due to incorporation of radionuclides inside the human body. Internal dosimetry calculations provide estimates of the amount of radiation that is absorbed by different organs or organ systems. Such assessments require several steps starting from monitoring, such as whole body counting or urinary excretion measurement, followed by application of biokinetic and dosimetric models and estimation of the exposure time, physical and chemical characteristic of radioactive substances, mode of intake, etc. [38]. Since the assessment technique involves many parameters consisting of the number of variable and uncertainties, the results may vary over a wide range depending on the experience of the assessor.

Assessment of intakes of radionuclides may be achieved by several techniques, i.e. direct measurement of activity in whole body or organs (*in vivo* counting); measurement of activity excreted with urine and feces (*in vitro* measurement); and personal air sampling and workplace monitoring. In some circumstances, a combination of these techniques may be applied to achieve an adequate sensitivity and accuracy. The IAEA has issued guidance on the direct measurement of body activity [39]. In principle, this assay can be carried out by using high efficiency detector housed in a well-shielded environment with low-background radiation. It is feasible only for radionuclides that emit high penetrating radiation, i.e. X- or gamma rays (e.g. ^131^I, ^137^Cs and ^60^Co), positron, energetic beta particles that can be detected by bremsstrahlung measurement (e.g. ^90^Y for ^90^Sr), and alpha emitters such as ^235^U and ^241^Am that can be detected by measurement if their characteristic gamma rays. The activity calibration is an important part of *in vivo* counting. It can be achieved by using the calibration phantoms with appropriate tissue substitutes. However, several physical phantoms have significant limitations regarding the body size, body shape and radionuclide distribution. Recently, these limitations have been overcome using the numerical calibration techniques based on medical image data [40] such as using mathematical voxel phantom constructed from CT or MRI for calibration of detectors in lung and knee counting geometry [41]. The calibration using voxel phantoms and Monte Carlo methods are very useful for the improvement in the accuracy of measurement results.

*In vitro* measurement using excreta monitoring is the other choice of measurement techniques. It is generally used for radionuclides that emit only low energy photon or alpha and beta particles. The specimens commonly used for *in vitro* measurement are urine and feces. However, for specific investigation, nose-blow and nasal smears might be used as routine screening techniques. Analysis of gamma-emitting radionuclides in excreta samples can be done by direct measurement with scintillation or semiconductor detectors, but analysis of radionuclides that emit alpha- and beta-rays requires chemical separation followed by appropriate measurement techniques such as alpha spectrometry, liquid scintillation counting, accelerator mass spectrometry (AMS) and inductively coupled plasma-mass spectrometry (ICP-MS).

Apart from these two measurement techniques, the personal air sampling and workplace monitoring are the alternative choices for assessment of intake of radionuclides. Personal air samplers (PAS) are commonly used to monitor activity concentration in air in the breathing zone of the workers, whereas static air samplers (SAS) are used to monitor workplace conditions. The use of PAS and SAS is the important part of a comprehensive workplace monitoring program as they can provide an early indication of risk of exposure.

Biokinetic and dosimetric models provide all data needed for dose calculation from incorporation of the radionuclides. Due to the complexity of calculation procedure, ICRP provided sets of dose per intake coefficients allowing a direct estimate of the internal dose from knowledge of body intake. The appropriate biokinetic and dosimetric models will be selected and applied for the assessment of internal dose from the monitoring data. There are many different approaches for the interpretation of monitoring results demonstrated earlier in ICRP Publication. The reliability of the dose assessment depends on the number and type of the monitoring data [38]. Some additional information might be considered as input parameters, such as time of intake, route of intake, aerosol size, GI tract absorption factor, etc. ICRP Publication 30 firstly demonstrated the general biokinetic model described in a form of compartmental model to calculate the effective dose [42]. Recently, biokinetic models have been updated for some elements such as the Human Respiratory Tract Model (HRTM) and the Human Alimentary Tract Model (HATM) described in ICRP Publication 66 and ICRP Publication 100 [43, 44]. ICRP has also continuously improved the accuracy of the biokinetic and dosimetric models. A new set of models and dose coefficients will be published in the Occupational Intake of Radionuclides (OIR) series and the Environmental Intakes of Radionuclides (EIR) series [45].

Internal dosimetry plays an important role in nuclear medicine as it is an essential tool in the calculation of radiation doses for radiopharmaceuticals. The calculated radiation doses is an essential element of balancing the risks and benefits of the proposed administration. Methods and tools exist for the calculation of radiation dose to important tissues of the body, especially for therapeutic applications. Dose estimates for diagnostic radiopharmaceuticals are not generally needed on a daily basis due to the doses are generally low. However, this might lead to misadministration if radiopharmaceutical is accidentally or intentionally administered to a pregnant woman, and in a few other limited circumstances [46]. Effective dose values for a number of diagnostic radiopharmaceuticals have been given in ICRP Publication 103 [47]. For therapeutic applications of radiopharmaceuticals, the maximum amount of the therapeutic agent can be determined from biokinetic data gathered for each patient by using a predictive dosimetry study derived from a diagnostic level of the therapeutic agent or surrogate. For instance, the biodistribution of a therapeutic agent, ^90^Y-Zevalin, can be determined from the use of diagnostic agent ^111^In-Zevalin [46].

Internal dose assessment has been carried out in many cases of the accidental intakes of radionuclides for decades. The recent extensive experience has been performed in an accident of internal contamination with plutonium (Pu) at a nuclear research institute in Japan. The accident occurred on 6 June 2017 at the Plutonium Fuel Research Facility (PFRF) of the Orari R&D Institute [48]. Five workers were contaminated with Pu compounds during operations to check storage containers including old Pu test samples for X-ray diffraction. These samples were put in doubled plastic bags, however, the bags raptured due to the gas generated by long-term alpha irradiation, resulting in external and internal contamination for the workers. Although external contamination found in all five workers, a positive nasal swab was detected in only three of them. The chelation therapy using calcium diethylenetriaminepenta-acetate (CaDTPA) was performed to remove inhaled Pu compounds for all of them including the two workers without a positive nasal swab. The bioassay results demonstrated the efficacy of chelating agent on a removal of Pu incorporated in the body. This case study is very useful to develop the DTPA model for the diagnosis of internal contamination even when a nasal swab is negative.

## FUTURE PROSPECTS OF BIOLOGICAL AND INTERNAL DOSIMETRY FOR MEDICINE

While biodosimetry using traditional cytogenetic assays are useful for dose assessment following accidental exposure to radiation, the field of biodosimetry has advanced significantly with expansion into the fields of genomics, proteomics, metabolomics and transcriptomics [49]. Advances in assessment of radiation response using those approaches have more relevant to clinical applications for patients undergoing radiation therapy. The information derived from radiation response may advance personalized care and could help mitigate normal tissue toxicity.

Currently, the science of internal dosimetry in nuclear medicine is well-developed. Many resources for performing dose calculations are available including internet and software tools [46]. Despite recent advances in the field of internal dosimetry, however, there are still technical and clinical limitations that hinder their applications in therapeutic planning. One of the main reasons is that radiopharmaceuticals administered in nuclear medicine are unsealed sources, consisting of a radioisotope combined with a chemical compound and exhibit extensive biodistribution in the organism due to their pharmacokinetic behavior which leads to the difficulty in determining their residence time in each organ or tissue. This results in the difficulty in determining the absorbed dose at the site of interests. It should be emphasized that the planning of radionuclide therapy in nuclear medicine is usually based on the radioactivity expressed in Becquerel (Bq) or Curie (Ci) administered to the patient, rather than the absorbed dose. However, the efficacy of radionuclide therapy are likely to be more directly related to absorbed doses in target tissues than to the administered radioactivity.

Due to the advancement of radiopharmaceuticals, new era in nuclear medicine will come up with theranostics, which is a combination of single drug used both for diagnostic and therapeutic purposes [50]. In addition to theranostics, a specific and personalized treatment to the patient is an emerging issue of interests. Individualized dose calculations are the most important for the patient, therefore, their relevant necessities are needed to be developed. These might include new biokinetic models for describing the behavior of radionuclides in human body; the individual phantom for a personalized set of dose coefficients; a new computer program for biokinetic modelling and internal dose calculations. The recent trend with an emerging targeted radionuclide therapy, in which new radiopharmaceuticals with beta- and alpha-emitters are approved in the clinical practice, the internal microdosimetry is needed to apply for internal dose assessment to patients at the cellular level [51].

## OUTCOME OF THE TRAINING MEETINGS

Learning outcomes of the educational discourse included an in-depth understanding of the standard classic techniques in biological and internal dosimetry, application of currently accepted techniques for dose estimation, comparison of advantages and weaknesses of standard techniques and evaluation of novel protocols in terms of accuracy and timeliness. The participants had extensive interactive sessions covering various aspects of biological and internal dosimetry as well as laboratory exercises. It included the detailed description of the currently available and widely accepted biodosimetry practices. In the domain of early-response approaches, it included measuring radioactivity and the clinical monitoring of the exposed individual, performing CBCs, carrying out cytogenetic bioassays aimed at different end-points, such as DCA, CB-MN, WCP FISH and PNA-FISH assays, among others for dose assessment in blood cells and measurement of free radicals in solid matrix materials using EPR [10, 15, 16]. The lectures delivered by experts in different techniques used for biodosimetry were very useful to the trainees who obtained a comprehensive view of the biodosimetry as well as its limitations. The description of nuclear incidents of the past, especially of the most recent one at Fukushima, and how they were dealt with, brought out to fore, the practical difficulties faced in performing biodosimetry under demanding conditions using conventional technologies for triage and medical interventions. The meetings also exposed the participants to the newer and novel emerging technologies in biological and internal dosimetry that can potentially overcome the limitations of existing and conventional technologies, particularly following large-magnitude nuclear incidents in the future. Notable among them were the envisioned use of molecular biomarkers and multi-parametric approach to biodosimetry. Besides these, each participant shared their own preparedness especially in terms of setting up or strengthening a cytogenetic biodosimetry laboratory in their respective countries, generating their own calibration curves and training manpower. Further information in the form of detailed presentations of the experts and the trainees of the 2013 training meeting are available at: https://humanhealth.iaea.org/HHW/RadiationOncology/RadiationBiology/Biological_Dosimetry/Biodose-21/index.html.

The academic discourse also laid the seed of a biodosimetry network for the Asia-Pacific region for mutual cooperation in strengthening biodosimetry laboratories across the region. This nascent initiative needs to be developed and formalized in the future so that regional networks may be formed and made operational to deal with regional nuclear catastrophes by mutual cooperation.

### AUTHORS’ NOTE

Some parts of this article draw on informal discussions that were held by a core of participants during the BIODOSE-21 Workshop in Hiroshima in June 2013. This core consisted of Rajeshwar N. Sharan, William F. Blakely, Prakash M. Hande, Yoshiaki Kodama, Tomisato Miura, Toshiteru Okubo, Eduardo Rosenblatt, Kazuo Sakai, Yumiko Suto, Kimio Tanaka, Koichi Tanigawa and Mitsuaki A. Yoshida.

## DISCLAIMER

The views expressed here are those of the authors and do not necessarily represent the official positions of either their respective institutions/organizations/universities/institutes or their governments.
